# The Efficacy of Defensive Antibacterial Coating (DAC™) Periprosthetic Joint Infection Prevention in the Hip: A Systematic Review

**DOI:** 10.3390/jcm14010270

**Published:** 2025-01-05

**Authors:** Antonio Bove, Adriano Braile, Giovanni Matino, Nicola Del Regno, Sabrina Sirico, Nicola Orabona, Mariantonia Braile

**Affiliations:** 1 Unit of Orthopaedics and Traumatology, Ospedale del Mare, 80147 Naples, Italy; antonio.bove@aslnapoli1centro.it (A.B.); adriano.braile@hotmail.it (A.B.); giovamat3@yahoo.it (G.M.); delregno.nicola@libero.it (N.D.R.); nicola.orabona@aslnapoli1centro.it (N.O.); 2 Department of Medical and Surgical Specialties and Dentistry, University of Campania “Luigi Vanvitelli”, 81100 Naples, Italy; 3 Department of Clinical Sciences and Translational Medicine, University of Tor Vergata, 00133 Rome, Italy; 4 Medifor, 80013 Naples, Italy; info@mediforsrl.it; 5 Department of Woman, Child and of General and Specialized Surgery, University of Campania “Luigi Vanvitelli”, 81100 Naples, Italy

**Keywords:** antibacterial hydrogel, arthroplasty, defensive antibacterial coating, periprosthetic joint infection

## Abstract

**Background:** Periprosthetic joint infections (PJIs) are a significant issue in joint replacement surgery patients, affecting results and mortality. Recent research focuses on developing hydrogels (HG) and antimicrobial coatings to reduce pressure injuries, with DAC™ HG showing lower infection risk in hip revision surgery. However, the effectiveness of DAC™ hydrogel in PIJs is still unknown. Here, we attempt to update the literature in this field, pointing out methodological flaws and providing guidance for further research. **Methods:** We conducted a systematic literature review using the PRISMA guidelines. Quality assessment was performed with the Newcastle–Ottawa Scale (NOS) and the Coleman Methodology Score (CMS). **Results:** Among 27 records from the initial search, 3 studies resulted eligible for final evaluation. It was observed that following the three surgical procedures performed in combination with DAC™ loaded with specific antibiotics, the quality of life of the treated patients had improved. No side effects associated with DAC™ treatment were in fact observed. **Conclusions:** The amount and quality of scientific evidence are yet insufficient to either encourage or dissuade the use of such hydrogels in hip prosthesis, despite some intriguing first results. These challenges will be better addressed by randomized controlled trials or longitudinal prospective investigations.

## 1. Introduction

Arthroplasty represents a successful elective surgical procedure for patients affected by arthritis, fractures, or oncological conditions with a survivorship of about 88% at more than 15 years of follow up [1]. When properly performed, this procedure enhances the quality of life of patients, providing pain relief and restoration of joint function [1].

Periprosthetic joint infections (PJIs) represent a challenging complication for a minority of patients undergoing surgery for joint replacement, and despite their low range of incidence, they represent a terrible event, compromising the results of the procedure and even increasing mortality by up to 20% [2]. PIJ is reported with an incidence ranging from 0.5 to 3% after primary arthroplasty of the hip or knee [3], resulting in the third greatest cause of revisions at 8.8–18.6%, and 22% of revision causes is associated with surgical re-operations [4]. This incidence grows to 0.5% to 15% when considering high-risk and oncological cases and in other conditions [5]. The bacteria most frequently responsible for a PJIs are *S. aureus* (60%) and *S. epidermidis* [6]. In detail, biofilms—a complex matrix of extracellular polymeric substances—are essential [7] to protect the bacterial growth on implant surfaces. There are four stages to their life cycle: attachment, proliferation, maturation, and emigration. The structure of the generated biofilms limits the penetration of antibiotics, impeding the removal of infection. In addition, they adhere to inorganic things, including joint replacement implants [8]. Treatments for PJIs, such as prolonged systemic antibiotic treatment and revision surgery in one or two stages, run the consequent risk of compromising functional outcomes and quality-of-life worsening [9], which represent a significant economic burden for national health systems. As proof of the fact, the management of patients with PJIs requires high financial resources, which is associated with great medical resource utilization and an increase in hospitalization costs, estimated at about seven times greater than in non-infected patients [10].

Several different efforts have been made to provide prevention strategies to avoid or significantly reduce PJI incidence after arthroplasty, but the management of PJIs still remains problematic and is associated with high financial burden and notable rates of morbidity and death [11]. In front of these problems, research has implemented efforts through the years in pre-, intra-, and post-operative fields [11]. In the pre-operative phase, prevention plays a crucial role as the first step in removing all the risk factors upstream. Over the years, several innovations have been developed in this context, including the application of antibacterial hydrogel (HG) during surgery [12,13]. HG is composed of hydrophilic building blocks, which could be cross-linked by physically reversible or chemically irreversible linkages to form three-dimensional space-spanning networks [14]. These devices reduce the incidence of PJIs by preventing bacterial adherence to implant surfaces and the formation of biofilms. In less than 72 h they completely release the antibiotic, after which they undergo hydrolytic disintegration [12,13,15].

Among these antibacterials, the HG known as Defensive Antibacterial Coating (DAC™—Novagenit, Mezzolombardo, Italy) is included. DAC™ is composed of bioresorbable polymers: hyaluronan, poly-D, and L-lactide [16,17,18]. It is produced as a powder, which must be hydrated with water before being used to form the hydrogel formulation for injectable preparations alone or in solution with an antibiotic [19]. As a matter of fact, this HG is able to form a physical barrier against bacterial adhesion by releasing high concentrations of antibiotics at the implant site and by undergoing complete hydrolytic degradation within 72 h [16,17,18,19,20]. Preclinical studies have demonstrated its ability to lessen bacterial colonization, exhibiting a favorable safety and efficacy profile [17,21]. Owing to these encouraging outcomes, DAC™ has been tested in several pilot studies regarding joint replacement, traumatology, and orthopedic oncology. For instance, in joint replacement [22,23,24], its application induces a reduction in infection occurrence in first implant hip replacement, in hip revision surgery [23,24], and in mega-prostheses implants [22]. Despite these efforts, the problem of efficacy of the HG DAC™ in PJIs is still unsolved. Indeed, although good results are reported in several studies, with no adverse events and a theorical strong impact on management of the patients, efficacy has to be proved [22,23,24].

This systematic review provides an overview of the available literature, trying to provide a critical revision to answer to the many questions surrounding the use of this device in prosthetic surgery (“Is DAC™ efficacious in preventing PJIs?”, “Is it safe and have a positive benefit/harms report?”) and indicating methodological deficiencies and perspective for future studies.

## 2. Methods

### 2.1. Information Sources and Search Strategy

A systematic review was performed to identify all studies reporting outcomes of DAC™ hydrogel application in primary and revision prosthetic surgery.

The studies have been searched in three different databases, Embase, Pubmed, and Scopus. The following keywords have been mixed in different combinations for each data bank: “Defensive antibacterial coating” or “Antibacterial hydrogel” or “Antimicrobial hydrogel” or “Antimicrobial hydrogel coating” or “DAC” and “arthroplasty” and “periprosthetic joint infection”. The research of databases ended on the 12th October 2024.

To assess the eligibility of each article, each PICO (Population, Intervention, Comparison, Outcome) element was identified as follows: Population (P): subjects (human) with PJI; Intervention (I): one-stage or two-stage hip revision surgery, and modular megaprosthesis; Comparison (C): presence/absence antibacterial HG treatment (DAC™); Outcome (O): diagnostic effectiveness of DAC™ methods to prevent PIJs after surgery.

The various topics analyzed in this systematic review are summarized in a checklist according to the Preferred Reporting Items for Systematic Reviews and Meta-Analyses (PRISMA) guidelines (PRISMA) 2020 Statement [25]. The PRISMA checklist is reported in the Supplementary File S1.

### 2.2. Eligibility Criteria

One author searched the combination of keywords using the Boolean operators AND and OR across three different databases. Afterwards, the author combined the three spreadsheets into a single spreadsheet, eliminating duplicates. Then, independently, three authors screened each remaining article based on the following inclusion criteria: (i) English language and (ii) studies that evaluate the efficacy of DAC™ in primary and revision prosthetic surgery.

The articles were evaluated as not eligible considering the following specific exclusion criteria: (i) articles missing one or more keywords, (ii) book chapter or note, (iii) conference abstract, (iv) in vitro study, (v) irrelevant articles to the main subject, (vi) no English language, (vii) and review or systematic review. The reasons for the exclusion of each record are reported in Supplementary File S2.

The title and abstract of all studies found in the search were independently examined by two reviewers who applied the eligibility criteria. In case of disagreement between the reviewers, a third reviewer was consulted.

### 2.3. Data Extraction and Quality Process

Following selection, data extraction was carried out by manual curation. The data were extracted by three authors who then independently summarized each article’s findings. Next, the “Methodological Index for Non-Randomized Studies” (MINORS) is a critical tool designed to evaluate the methodological quality of non-randomized studies, particularly in the field of clinical and epidemiological research [26]. This assessment method comprises a 12-item checklist that helps researchers and clinicians systematically assess key aspects of study design and execution. The criteria include elements such as the clarity of the study aim, the inclusion of consecutive patients, the prospective collection of data, and the definition of endpoints. Additionally, MINORS evaluates the assessment of outcomes, follow-up periods, and any potential biases in selection and classification. Each criterion is scored as “Yes” or “No”, allowing for a straightforward evaluation of the overall quality. By providing a structured framework for assessing methodological rigor, the MINORS tool aids in identifying the strengths and weaknesses of non-randomized studies, ultimately contributing to more reliable and evidence-based interpretations of research findings [26].

At last, a modified version of the “Coleman Methodology Score” (mCMS) (Supplementary File S3) was used to assess the methodological quality of observational studies in medical research [27]. A score between 0 and 100 was assigned, with 100 denoting a study that fully avoids the influence of chance, different biases, and confounding factors. Every reviewer completed the evaluation twice, separated by ten days. Supplementary File S3 contains the total number of Coleman scores as well as the scores for each of the ten Coleman criteria [27].

## 3. Results

### 3.1. Literature Research

**Figure 1:**
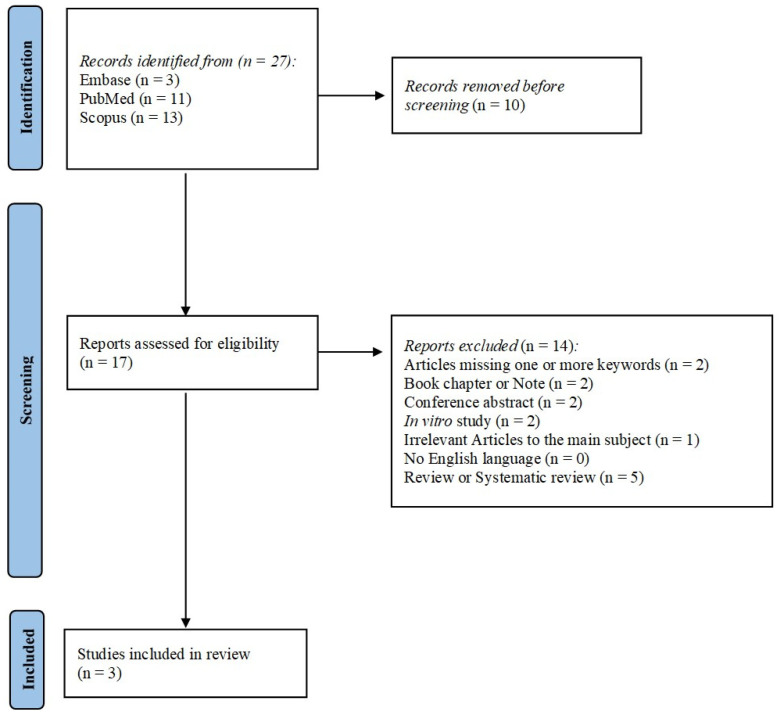
PRISMA flow-diagram showing research strategy [25].

The flowchart provides a clear illustration of the process for literature searching (Figure 1). A total of 27 articles were obtained by interrogating PubMed, Embase, and Scopus based on the following key words: “Antibacterial hydrogel”, “Antimicrobial hydrogel coating”, “Antimicrobial hydrogel”, “Arthroplasty”, “Defensive antibacterial coating”, and “Periprosthetic joint infection” After discarding duplicates, 17 articles were assessed according to the eligibility criteria. After completing the screening procedure, three articles were included in this systematic review in order to elucidate the effectiveness of DAC™ in preventing hip PJI.

### 3.2. Study Characteristics

The main characteristics of all the included studies are reported in Table 1. All the included studies were published between 2019 and 2021 and were conducted on human subjects. All the selected studies are retrospective [22,23,24]. Two of these are case controls [22,24], of which only one is a multicenter study [22]. The number of subjects enrolled in the studies varied from 10 to 86. All included studies required the presence of PJI, a surgical operation, a follow up of at least 1 year, and antibiotic prophylaxis [22,23,24]. Based on the outcomes of each study, we analyzed the effects of DAC™ on PJI in association with the operation performed: one, two-stage, and the modular megaprosthesis procedure.

### 3.3. Clinical Outcome

#### 3.3.1. One-Stage Hip Revision Surgery

In Pellegrini et al. (2021) manuscript [23], patients with PJI underwent total hip arthroplasty using the one-stage approach with DAC™-coated implants plus antibiotics (Table 1). As minutely described, 3.1 years on average (range, 2–5) of follow-up, there were no radiographic or clinical indicators of recurrent infection (0/10). Scores for pain and functionality were both greatly enhanced. The mean HHS improved from an average pre-operative value of 38.1 points (SD: 10.3) to 81.3 points (SD: 6.7) of post-operative value. Visual analog scale improved from 6.8 (SD: 3.2) to 1.9 (SD: 2.4). The bacteria isolated at the time of surgery were coagulase-negative Staphylococci and methicillin-resistant *Staphylococcus aureus* (MRSA). Following treatment with DAC™ loaded with specific antibiotics (Table 1), the bacterial infection was completely eradicated in all patients.

Taken together, data shows that the one-stage revision, combined with antibacterial HG-coated implants, for patients with PJI, it is an accurate and reliable technique that prevents infection and yields subjectively acceptable functional outcomes [23].

#### 3.3.2. Two-Stage Hip Revision Surgery

The current investigations were carried out to test the assumption that a two-stage cementless revision of an infected hip prosthesis using implants coated with DAC™ could yield better outcomes and a lower rate of reinfection than a two-stage revision carried out without the coating [24].

According to Zagra et al. (2019) [24], first-stage surgery included removing the contaminated prosthesis, debridement of the soft tissues and infected bone, eliminating foreign bodies by means of a standardized surgical technique, and inserting a spacer that was loaded with antibiotics. This antibiotic-loaded spacer was extracted during the second treatment, which involved inserting the definitive implant. Another debridement of the soft tissues and bone was conducted. Following sufficient preparation, a prosthetic cementless implant was inserted. At a follow-up period of 2.7 ± 0.6 years, the control and DAC™-treated groups had HHS of 81.6 ± 15.2 and 84.6 ± 15.8, respectively. Infections did not occur in the DAC group, whereas four infection cases occurred in the control group (two *S. capitis*, one MRSA, and two *Staphylococcus epidermidis*). There were no adverse events that could be directly linked to the DAC™ HG, either locally or systemically. Neither radiological nor implant loosening or subsidence were observed [24]. The authors demonstrate that a two-stage procedure with uncemented prostheses and easily resorbable DAC™ loaded with antibiotics could be effective in managing patients with PJI while avoiding side effects.

#### 3.3.3. Modular Megaprosthesis

In Zoccali et al. (2021) [22], every patient was fitted with a mega-prosthetic device. Interestingly, this study further divided patients and controls into oncology and non-oncology. The results showed that compared to the treated group, which had no infections, 13.9% of control patients had post-surgical infections. After reconstructing and resecting the extremities, 8.1% of patients in the control group had an infection at the surgical site, and 50% had pelvic resection and implant placement. Surgical debridement, antibiotic therapy, implant revision, and limb amputation were used to treat mega-implant infections. 15 days following surgery, there was wound dehiscence in the treated group; however, there were no signs of deep or organ space infections or implant revision as a result of septic sequelae. 6 patients showed progression of the oncological disease, necessitating revision of implants, amputation of limbs, and death from tumor recurrence. No adverse events related to the use of the antibacterial HG were reported [22].

### 3.4. Quality Assessment and Risk of Bias Across Studies

The application of the MINORS tool to the selected studies on the use of DAC™ reveals varying strengths and weaknesses in their methodological quality (Table 2) [26]. Zagra et al. (2019) [24] demonstrated strong characteristics with clearly stated aims and defined endpoints, as well as a good follow-up period, but fell short in terms of prospective data collection and the absence of sample size calculations. Similarly, Zoccali et al. (2021) [22] highlighted clear endpoint definitions and unbiased assessment of outcomes; however, it was limited by not including consecutive patients and also lacked a sample size calculation, which could potentially affect the power of the study. Lastly, Pellegrini et al. (2022) [23] exhibited solid methodological attributes, notably in endpoint clarity and follow-up, but reported no loss to follow-up and did not perform sample size calculations. In summary, while all three studies present valuable insights into the efficacy of DAC™ in preventing infections, addressing the identified methodological limitations—particularly the need for prospective data collection and appropriate sample size calculations—would enhance the robustness of the evidence, leading to more reliable conclusions in future research.

### 3.5. mCMS

In Table 3 it has been shown the summary of three retrospective cohort studies focused on surgical interventions for joint prosthesis revision and infection management. These retrospective analyses offer insight into different surgical approaches, with the Modified Coleman Methodology Score (mCMS) used to assess the quality of each study (ranging from 29 to 46) [27]. This mCMS evaluates several key factors, including sample size, follow-up duration, and the percentage of patients followed up with both radiographic and clinical assessments. Additional criteria assess the number of surgical procedures included in each study, the type of study (with higher scores assigned to randomized controlled trials), and diagnostic certainty based on standardized clinical and radiographic evaluations. Furthermore, our CMS takes into account the thoroughness in describing surgical procedures and postoperative protocols, as well as the clarity and reliability of outcome criteria. The scoring also reflects whether an independent investigator conducted the outcome assessments. The Supplementary File S3 underlines the critical role of standardized methods in ensuring reliable and reproducible study outcomes in orthopedic surgery. In detail, Table 3 provides a breakdown of mCMS scores for these selected studies, highlighting key strengths and limitations in their methodologies. None of the studies has been considered Excellent, Good, or Moderate, while all the studies received a score < 50, resulting as “Poor” quality studies [27].

The research conducted by Zoccali et al. [22] about antibacterial hydrogel coatings for joint mega-prostheses received the highest mCMS score (46 in total), largely due to its large sample size, extensive follow-up, and well-documented postoperative protocol. However, the study scored lower on the assessment of different surgical procedures and the lack of a standardized diagnostic flowchart.

The study by Pellegrini et al. [23] showed merits in its precisely specified surgical techniques and outcome measurements while having a lower overall score (43). However, it was limited by a small sample size (10 patients) and a lack of detailed reporting on some key diagnostic and selection processes.

Lastly, the article of Zagra et al. [24] on two-stage cementless hip revision surgery had the lowest mCMS score [27], particularly due to incomplete reporting on patient follow-up and surgical outcomes, as well as a less detailed postoperative protocol. Despite these limitations, the study did provide adequate diagnostic certainty.

## 4. Discussion

This systematic review aimed to clarify the efficacy of DAC™ on PJI prevention in the hip. Following PRISMA 2020 standards, this systematic review was performed analyzing electronic databases in order to compile pertinent articles. After arthroplasty, PJI continues to be one of the most difficult and debilitating side effects. Research on biomaterials, surgical techniques, diagnostic tools, and proactive measures are aimed at reducing PJIs following arthroplasty [18], such as DAC™. DAC™ is an antibiotic-loaded HG used in clinical trials, i.e., in the first implant hip replacement and in hip revision surgery and in megaprosthesis implants. Here, we discussed the effect of DAC™ treatment on patients with PJI after revision surgery [23,24] and the megaprosthesis procedure [22].

In the one-stage revision surgery [23], the perks consist of the patients only requiring one main treatment, which saves hospital stays and expenses by preventing issues related to the use of temporary spacers, such as spacer dislocation or antibiotic allergy reactions. Nevertheless, preoperative identification of the infectious organism and its sensitivity is a critical prerequisite for one-stage exchange arthroplasty [28,29]. In addition, the retrospective design of the Pellegrini et al. study, the limited sample size, and the lack of a control group are some of its drawbacks.

In the Zagra et al. article [24], it has been demonstrated that a fast-resorbable antibacterial HG covering can lower the rate of reinfection following two stages of cementless revision surgery while having no discernible negative effects. Less time spent in the hospital was another finding in the group of patients receiving DAC™ treatment. Even though this effect is dubious, it is possible that a quicker and better recovery would have a favorable impact on expenses as well as patient recovery. Even in this article, a number of flaws are clear, including the limited sample size, the brief follow-up period—which was, however, sufficient to rule out HG side effects—the lack of implant durability, and the possibility of reinfection. An additional restriction is the absence of a systematic antibiotic treatment protocol.

Following significant resection of bone and soft tissue cancers, PJIs remain a critical concern in mega-prosthetic reconstructions [30]. Zoccali et al.’s study [22] indicates that patients with hydrogel (HG)-coated mega-implants experience a statistically significant incidence of surgical site infections, despite a lack of adverse events. The limitations, including low patient enrollment and short-term follow-up, challenge the conclusions regarding the HG coating’s effectiveness.

In contrast, among the new approaches presented, a reconstruction technique that uses vancomycin-containing cement to provide localized antimicrobial treatment is interesting [30]. While promising, it raises concerns about antibiotic resistance. Positive aspects of the DAC™ method include its dual action of preventing bacterial adhesion while releasing antibiotics in a controlled manner. This may reduce systemic side effects and toxicity compared to traditional antibiotics. Overall, while both studies address infection prevention, the DAC™-coated implants present an innovative strategy that could improve outcomes in high-risk patients undergoing complex reconstructions.

However, all these findings are primarily based on a series of case–control studies, which, while consistent, lend themselves to a Grade C recommendation according to Guyatt et al. [31]. This classification indicates a weak strength of recommendation due to the lack of comprehensive evaluations assessing the risk–benefit and cost–utility ratios of DAC™ compared to other therapeutic strategies. Therefore, further rigorous studies are warranted to substantiate its effectiveness and determine its place in the broader context of infection management.

Furthermore, the articles that emerged from our search were all conducted retrospectively. Nevertheless, part of our results are clearly confirmed by other studies, such as from both the group of Malizos et al. [32] and the group of Capuano et al. [33], where no adverse events were highlighted and the number of reinfections after surgery in patients treated with DAC™ compared to the control group was reduced or non-existent. More information may come upon closure and data publications of the prospective open-label randomized clinical trial currently studying DAC™ (ID NCT04251377).

In general, to accurately determine the effects of DAC™ combined with the three types of procedures on PJI patients, further studies performed on a large scale, with long-term follow-up and with a standardized treatment protocol are needed. Additionally, it is important to acknowledge that the current research on DAC™ is primarily limited to Italy. This geographic limitation highlights the necessity for further international studies to validate the efficacy and safety of this product across diverse populations and clinical settings. Broadening the scope of research will be essential in establishing a robust global understanding of DAC™’s applications in preventing PJI.

## 5. Conclusions

In conclusion, although there are initial positive findings, the quality and quantity of scientific evidence are still too limited to either support or discourage the use of such HG in hip prostheses. Longitudinal prospective studies or randomized controlled trials will better answer these questions.

## Figures and Tables

**Table 1:** Main characteristics of the selected studies on PJI patients treated with DAC™ loaded with antibiotics and surgery procedures.

Authors	Study Design	Enrolled Population, *n*	Surgical Procedures	Follow up	Antibiotic Prophylaxis	PJI Eradication, %	Clinical Parameters Evaluated
Pellegrini et al. (2021) [23]	Retrospective Study	10 patients	Cementless one-stage hip revision surgery with DAC™ loaded with a combination of Vancomycin and Gentamicin	3.1 years (20–26 months)	6–8 weeks, starting with Vancomycin 1 g two times a day and Ciprofloxacin 400 mg two times a day At discharge, intravenous therapy was converted to targeted oral therapy according to specific microorganism isolation	100%	HHS VAS pain score Radiographic examination
Zagra et al. (2019) [24]	Retrospective, Comparative Study	54 in total: 27 patients undergoing DAC™-coated 27 controls without the DAC™ coating	Cementless two-stage hip revision surgery with DAC™ loaded with Vancomycin, Teicoplanin, Ceftazidime, Rifampicin, Meropenem	2.7 ± 0.6 years (min. 2, max 3.5)	4–6-week antibiotic therapy, starting with Vancomycin 1 g bid and meropenem 1 g tid, then switched to targeted oral therapy based on intraoperative culture After the surgery, systemic antibiotic therapy was continued until the results of intra-operative cultures, and for a minimum of two weeks post-operatively Specific antibiotic therapy continued until the results of intra-operative cultures, for a minimum of 2 weeks	100% in the DAC group 14.8% in the control group	HHS Any sign of infection at the site of surgery Radiographic examination
Zoccali et al. (2021) [22]	Multi-center, Retrospective, Comparative Study	86 in total: 43 patients undergoing DAC™-coated, further divided into 39 with oncological pathology and 4 without oncological pathology 43 controls without the DAC™ coating, further divided into 39 with oncological pathology and 4 without oncological pathology	Cementic modular megaprosthesis With DAC™ loaded with Gentamicin, Vancomycin, Tobramycin	24.3 ± 11.7 months for the control group 24.2 ± 11.5 in the DAC group	Systemic antibiotic prophylaxis in all patients at the time of surgery and postoperatively for 6.5 ± 3.9 days (range 2–28) in the treated cohort and for 6.4 ± 4.0 days (range 3–28) in the control group	100% in the DAC group 6% in the control group	Any sign of infection at the site of surgery Radiographic examination

Abbreviation: DAC, Defensive antibacterial coating; HHS, Harris hip score; VAS, visual analog scale.

**Table 2:** MINORS Quality Assessment.

MINORS Criteria	Zoccali et al. (2021) [22]	Pellegrini et al. (2021) [23]	Zagra et al. (2019) [24]
1. A clearly stated aim	Yes	Yes	Yes
2. Inclusion of consecutive patients	Yes	No	Yes
3. Prospective collection of data	No	No	No
4. Endpoints that are clearly defined	Yes	Yes	Yes
5. Unbiased assessment of endpoints	Yes	Yes	Yes
6. Follow-up period appropriate to the intervention	Yes	Yes	Yes
7. Loss to follow up reported	Yes	Yes	No
8. Comparative groups are well defined	Yes	Yes	Yes
9. Statistical analysis used to assess the main outcome	Yes	Yes	Yes
10. A sample size calculation was performed	No	No	No
11. The study was multi-center	No	No	No
12. The study was funded by a relevant funding source	No	No	Yes

**Table 3:** mCMS applied to the studies included in the review.

Authors	Enrolled Population, *n*	Follow up (Mean)	Surgical Procedures	Study Design	mCMS
Zoccali et al. (2021) [22]	86	24.3	Primary hip and knee mega implants prosthetic surgery	Retrospective cohort study	46
Pellegrini et al. (2021) [23]	10	36	Septic hip “one stage” revision surgery	Retrospective cohort study	43
Zagra et al. (2019) [24]	54	31	Septic two stage cementless hip revision surgery	Retrospective cohort study	29

Abbreviation: mCMS, modified Coleman Methodology Score.

## Data Availability

All data generated or analyzed during this study are included in this published article and its Supplementary Information Files.

## Supplementary Materials

The following supporting information can be downloaded at: https://www.mdpi.com/article/10.3390/jcm14010270/s1. Supplementary File S1. PRISMA Checklist. Supplementary File S2. List of studies not included and exclusion reasons. Supplementary File S3. Modified Coleman Methodology.

## List of Abbreviations

CMS	Coleman Methodology Score
DAC™	Defensive Antibacterial Coating
HG	Hydrogel
HHS	Harris hip score
MINORS	Methodological Index for Non-Randomized Studies
MRSA	Methicillin-resistant *Staphylococcus aureus*
PJI	Periprosthetic joint infections
