# Supplementary material for: The Efficacy of Defensive Antibacterial Coating (DAC™) Periprosthetic Joint Infection Prevention in the Hip: A Systematic Review
Source: J Clin Med. 2025 Jan 5;14(1):270. doi: 10.3390/jcm14010270 (PMC11722025; doi:10.3390/jcm14010270)
Supplement: Supplementary file 1 [file jcm-14-00270-s001.zip › jcm-3383303-Supplementary.pdf]

Supplementary File S1

PRISMA 2020 Checklist

Section and Topic	Item #	Checklist item	Location where item is reported
**TITLE**			
Title	1	Identify the report as a systematic review.	Lane 2-3, page 1
**ABSTRACT**			
Abstract	2	See the PRISMA 2020 for Abstracts checklist.	Done
**INTRODUCTION**			
Rationale	3	Describe the rationale for the review in the context of existing knowledge.	Lane 38-92, pages 1-2
Objectives	4	Provide an explicit statement of the objective(s) or question(s) the review addresses.	Lane 93-98, page 2
**METHODS**			
Eligibility criteria	5	Specify the inclusion and exclusion criteria for the review and how studies were grouped for the syntheses.	Lane 127-131, page 3
Information sources	6	Specify all databases, registers, websites, organisations, reference lists and other sources searched or consulted to identify studies. Specify the date when each source was last searched or consulted.	Lane 103-109,page 3
Search strategy	7	Present the full search strategies for all databases, registers and websites, including any filters and limits used.	Lane 110-119, page 3
Selection process	8	Specify the methods used to decide whether a study met the inclusion criteria of the review, including how many reviewers screened each record and each report retrieved, whether they worked independently, and if applicable, details of automation tools used in the process.	Lane 121-126, Page 3;Lane 133-134, page 4
Data collection process	9	Specify the methods used to collect data from reports, including how many reviewers collected data from each report, whether they worked independently, any processes for obtaining or confirming data from study investigators, and if applicable, details of automation tools used in the process.	Lane 137-139, page 4
Data items	10a	List and define all outcomes for which data were sought. Specify whether all results that were compatible with each outcome domain in each study were sought (e.g. for all measures, time points, analyses), and if not, the methods used to decide which results to collect.	Lane 170-173, page 5
	10b	List and define all other variables for which data were sought (e.g. participant and intervention characteristics, funding sources). Describe any assumptions made about any missing or unclear information.	Lane 173-177, page 5
Study risk of bias assessment	11	Specify the methods used to assess risk of bias in the included studies, including details of the tool(s) used, how many reviewers assessed each study and whether they worked independently, and if applicable, details of automation tools used in the process.	Lane 139-144, page 4
Effect measures	12	Specify for each outcome the effect measure(s) (e.g. risk ratio, mean difference) used in the synthesis or presentation of results.	n.a.

PRISMA 2020 Checklist

Section and Topic	Item #	Checklist item	Location where item is reported
Synthesis methods	13a	Describe the processes used to decide which studies were eligible for each synthesis (e.g. tabulating the study intervention characteristics and comparing against the planned groups for each synthesis (item #5)).	Table 1
	13b	Describe any methods required to prepare the data for presentation or synthesis, such as handling of missing summary statistics, or data conversions.	n.a.
	13c	Describe any methods used to tabulate or visually display results of individual studies and syntheses.	Table1
	13d	Describe any methods used to synthesize results and provide a rationale for the choice(s). If meta-analysis was performed, describe the model(s), method(s) to identify the presence and extent of statistical heterogeneity, and software package(s) used.	Table 2
	13e	Describe any methods used to explore possible causes of heterogeneity among study results (e.g. subgroup analysis, meta-regression).	n.a.
	13f	Describe any sensitivity analyses conducted to assess robustness of the synthesized results.	n.a.
Reporting bias assessment	14	Describe any methods used to assess risk of bias due to missing results in a synthesis (arising from reporting biases).	Lane 252-269, page 9
Certainty assessment	15	Describe any methods used to assess certainty (or confidence) in the body of evidence for an outcome.	n.a.
**RESULTS**			
Study selection	16a	Describe the results of the search and selection process, from the number of records identified in the search to the number of studies included in the review, ideally using a flow diagram.	Figure 1
	16b	Cite studies that might appear to meet the inclusion criteria, but which were excluded, and explain why they were excluded.	Lane 153-157, page 4
Study characteristics	17	Cite each included study and present its characteristics.	Table 1
Risk of bias in studies	18	Present assessments of risk of bias for each included study.	Lane 270-282, page9
Results of individual studies	19	For all outcomes, present, for each study: (a) summary statistics for each group (where appropriate) and (b) an effect estimate and its precision (e.g. confidence/credible interval), ideally using structured tables or plots.	Table 1<br>Table 2
Results of syntheses	20a	For each synthesis, briefly summarise the characteristics and risk of bias among contributing studies.	Table 2<br>Supplementary 3
	20b	Present results of all statistical syntheses conducted. If meta-analysis was done, present for each the summary estimate and its precision (e.g. confidence/credible interval) and measures of statistical heterogeneity. If comparing groups, describe the direction of the effect.	Table 2<br>Supplementary 3
	20c	Present results of all investigations of possible causes of heterogeneity among study results.	Table 2<br>Supplementary

PRISMA 2020 Checklist

Section and Topic	Item #	Checklist item	Location where item is reported
			3
	20d	Present results of all sensitivity analyses conducted to assess the robustness of the synthesized results.	Table 2 Supplementary 3
Reporting biases	21	Present assessments of risk of bias due to missing results (arising from reporting biases) for each synthesis assessed.	Supplementary 3
Certainty of evidence	22	Present assessments of certainty (or confidence) in the body of evidence for each outcome assessed.	Supplementary 3
**DISCUSSION**			
Discussion	23a	Provide a general interpretation of the results in the context of other evidence.	Lane 308-316, page 10
	23b	Discuss any limitations of the evidence included in the review.	Lane 346-348, page 11
	23c	Discuss any limitations of the review processes used.	Lane 350-354, page 11
	23d	Discuss implications of the results for practice, policy, and future research.	Lane 350-354, page 11
**OTHER INFORMATION**			
Registration and protocol	24a	Provide registration information for the review, including register name and registration number, or state that the review was not registered.	n.a.
	24b	Indicate where the review protocol can be accessed, or state that a protocol was not prepared.	n.a.
	24c	Describe and explain any amendments to information provided at registration or in the protocol.	n.a.
Support	25	Describe sources of financial or non-financial support for the review, and the role of the funders or sponsors in the review.	Lane 379, page 11
Competing interests	26	Declare any competing interests of review authors.	Lane 386,
Availability of data, code and other materials	27	Report which of the following are publicly available and where they can be found: template data collection forms; data extracted from included studies; data used for all analyses; analytic code; any other materials used in the review.	n.a.

From: Page MJ, McKenzie JE, Bossuyt PM, Boutron I, Hoffmann TC, Mulrow CD, et al. The PRISMA 2020 statement: an updated guideline for reporting systematic reviews. BMJ 2021;372:n71. doi: 10.1136/bmj.n71

PRISMA 2020 for Abstracts Checklist

Section and Topic	Item #	Checklist item	Reported (Yes/No)
**TITLE**			
Title	1	Identify the report as a systematic review.	yes
**BACKGROUND**			
Objectives	2	Provide an explicit statement of the main objective(s) or question(s) the review addresses.	yes
**METHODS**			
Eligibility criteria	3	Specify the inclusion and exclusion criteria for the review.	no
Information sources	4	Specify the information sources (e.g. databases, registers) used to identify studies and the date when each was last searched.	yes
Risk of bias	5	Specify the methods used to assess risk of bias in the included studies.	no
Synthesis of results	6	Specify the methods used to present and synthesise results.	yes
**RESULTS**			
Included studies	7	Give the total number of included studies and participants and summarise relevant characteristics of studies.	yes
Synthesis of results	8	Present results for main outcomes, preferably indicating the number of included studies and participants for each. If meta-analysis was done, report the summary estimate and confidence/credible interval. If comparing groups, indicate the direction of the effect (i.e. which group is favoured).	no
**DISCUSSION**			
Limitations of evidence	9	Provide a brief summary of the limitations of the evidence included in the review (e.g. study risk of bias, inconsistency and imprecision).	yes
Interpretation	10	Provide a general interpretation of the results and important implications.	yes
**OTHER**			
Funding	11	Specify the primary source of funding for the review.	no
Registration	12	Provide the register name and registration number.	no

From: Page MJ, McKenzie JE, Bossuyt PM, Boutron I, Hoffmann TC, Mulrow CD, et al. The PRISMA 2020 statement: an updated guideline for reporting systematic reviews. BMJ 2021;372:n71. doi: 10.1136/bmj.n71

Supplementary File S2 List of articles excluded via eligible criteria application.

Authors	Title	Year	Journal	DOI	Exclusion Criteria
Aboltins CA *et al*	Hip and Knee Section, Prevention, Prosthesis Factors: Proceedings of International Consensus on Orthopedic Infections	2019	Journal of Arthroplasty	10.1016/j.arth.2018.09.016	Note
Barros J *et al*	Bioengineering Approaches to Fight against Orthopedic Biomaterials Related-Infections	2022	International Journal of Molecular Sciences	10.3390/ijms231911658	Review
De Martino I *et al*	Local Delivery of Antibiotic and Antiseptic	2021	Infection in Knee Replacement	10.1007/978-3-030-81553-0_13	Book chapter
Ghirardelli S *et al*	Debridement, antibiotic, pearls, irrigation and retention of the implant and other local strategies on hip periprosthetic joint infections	2022	Minerva Orthopedics	10.23736/S2784-8469.21.04173-0	No keyword
Levack AE *et al*	Current Options and Emerging Biomaterials for Periprosthetic Joint Infection	2018	Current Rheumatology Reports	10.1007/s11926-018-0742-4	Review
Li Z	Engineering Multifunctional Hydrogel-Integrated 3D Printed Bioactive Prosthetic Interfaces for Osteoporotic Osseointegration	2022	Advanced Healthcare Materials	10.1002/adhm.202102535	No keyword
Onorato F *et al*	What to Know about Antimicrobial Coatings in Arthroplasty: A Narrative Review	2024	Medicina (Lithuania)	10.3390/medicina60040574"	Review
Sille IE *et al*	Antimicrobial-Loaded Polyacrylamide Hydrogels Supported on Titanium as Reservoir for Local Drug Delivery	2023	Pathogens	10.3390/pathogens12020202	*In vitro* study
Pressato D *et al*	The Intraoperative Use of Defensive Antibacterial Coating (DAC®) in the Form of a Gel to Prevent Peri-Implant Infections in Orthopaedic Surgery: A Clinical Narrative Review	2023	Materials	10.3390/ma16155304"	Review
Pressato D *et al*	DACÂ® gel a hyaluronan based hydrogel antibiotic-loaded against biofilm formation: New clinical perspective in the prevention of periprosthetic joint infection	2017	Journal of Applied Biomaterials and Functional Materials	10.5301/jabfm.5000360	Conference abstract

Trentinaglia MT *et al*	Economic Evaluation of Antibacterial Coatings on Healthcare Costs in First Year Following Total Joint Arthroplasty	2018	J Arthroplasty.	10.1016/j.arth.2018.01.057	Irrelevant Articles to the main subject
Tschon M *et al*	Use of Antibiotic Loaded Biomaterials for the Management of Bone Prosthesis Infections: Rationale and Limits	2019	Curr Med Chem.	10.2174/0929867325666171129220031	Review
Tsikopoulos K *et al*	Is Implant Coating with Tyrosol- and Antibiotic-loaded Hydrogel Effective in Reducing Cutibacterium (Propionibacterium) acnes Biofilm	2019	Clinical Orthopaedics and Related Research	10.1097/CORR.0000000000000663	*In vitro* study
Wadgaonkar M *et al*	CO149 Effectiveness of Surface Modified Antimicrobial Implants in Treatment or Prevention of Periprosthetic Joint Infections Post Knee Replacement Surgery: A Systematic Literature Review	2022	Value in Health	10.1016/j.jval.2022.04.243	Conference abstract

Supplementary File S3 Modified version of the "Coleman Methodology Score".

**Study Size (n. of cases)**	N > 100<br><br>N 81 – 99<br><br>N 40 – 80<br><br>N < 40	10<br><br>7<br><br>4<br><br>0
**Mean follow up (months)**	> 24<br><br>12 – 24<br><br>< 12	5<br><br>3<br><br>0
**Percent of patients with follow up**<br><br>(Radiographic and clinical)	> 90%<br><br>80 – 90%<br><br>< 80% or not reported	5<br><br>3<br><br>0
**Number of different surgical procedures included in each reported outcome.**<br><br>More than one surgical technique may be assessed but separate outcomes should be reported	One surgical procedure only<br><br>More than one S.P. >90% of subjects undergoing the one procedure<br><br>Not stated, unclear or <90% of subjects undergoing the one procedure	10<br><br>7<br><br>0
**Type of study**	Randomised control trial<br><br>Prospective cohort study<br><br>Retrospective cohort study	15<br><br>10<br><br>0
**Diagnostic certainty**<br><br>Assessment according to standardized clinical criteria, radiographic evaluation and laboratory examinations including, in case of PJI suspicion: arthrocentesis after 2 weeks antibiotic suspension, white blood cell count and percentage of neutrophilic granulocytes ad sonication or chemical dithiothreitol perioperative analysis in case of implant removal.	Adequate diagnostic flowchart<br><br>Flowchart missing (or not specified) necessary diagnostic steps<br><br>Unadequate flowchart, not specified or unclear	5<br><br>3<br><br>0
**Description of surgical procedure**	Adequate (technique stated and necessary details of that type of procedure given<br><br>Fair (technique only stated without elaboration)<br><br>Inadequate, not stated or unclear	5<br><br>3<br><br>0
**Description of Postoperative Protocol**	Well described with >80% of patients complying	5

	Well described with 60 – 80% of patients complying<br><br>Protocol not reported or 60-80% of patients complying	3<br><br>0
**Outcome criteria**<br><br>Radiological evaluation reporting signs of focal osteolysis around the implant, signs of implant loosening or subsidence; laboratory results to confirm absence of infection; standardized clinical assessment	Outcomes measures clearly defined<br><br>Timing of outcome assessment clearly stated<br><br>Use of outcome criteria that has reported good reliability<br><br>Use of outcome with good predictive value	2<br><br>2<br><br>3<br><br>3
**Procedure for assessing outcomes**	Subjects recruited<br><br>Independent investigator<br><br>Written assessment<br><br>Completion of assessment by subjects themselves with minimal investigator assistance	5<br><br>4<br><br>3<br><br>3
**Description of subject selection process**	Selection criteria reported and unbiased<br><br>Recruitment rate reported >80%<br><br><80%<br><br>Eligible subjects not included in the study satisfactorily accounted for or 100% recruitment	5<br><br>5<br><br>3<br><br>5
